# Protocol for developing shape-morphing 4D bioprinted magnetic constructs to promote articular cartilage regeneration using silk fibroin-gelatin bioink

**DOI:** 10.1016/j.xpro.2024.103332

**Published:** 2024-10-23

**Authors:** Juhi Chakraborty, Chandrashish Roy, Maria Kalogeropoulou, Carlos Mota, Sourabh Ghosh, Lorenzo Moroni

**Affiliations:** 1 Department of Textile and Fibre Engineering, Indian Institute of Technology Delhi, New Delhi 110016, India; 2 Department of Complex Tissue Regeneration, MERLN Institute for Technology-Inspired Regenerative Medicine, Maastricht University, 6211 LK Maastricht, the Netherlands

**Keywords:** Stem Cells, Tissue Engineering, Material sciences

## Abstract

External magnetic fields can regulate cellular responses. Here, we present a protocol to fabricate magnetic constructs by 4D bioprinting with shape-morphing properties using silk fibroin-gelatin bioinks for articular cartilage regeneration. We illustrate the steps for magnetic bioink formulation, bioprinting, and chondrogenic induction of human bone marrow mesenchymal stem/stromal cells. We detail the steps to actuate the constructs using an external magnetic field and then characterize chondrogenesis. Magnetic field actuation may be helpful for mechanically activating constructs for articular cartilage regeneration.

For complete details on the use and execution of this protocol, please refer to Chakraborty et al.^1^

## Highlights

• Protocol for 4D bioprinting shape-morphing magnetic constructs
• Steps for forming bioink using silk fibroin-gelatin and magnetic nanoparticles
• Instructions for actuating magnetic constructs by an external magnetic field
• Details on molecular characterization of chondrogenesis

## Abstract (editor highlights)

Publisher’s note: Undertaking any experimental protocol requires adherence to local institutional guidelines for laboratory safety and ethics.

## Graphical abstract

**Figure:**
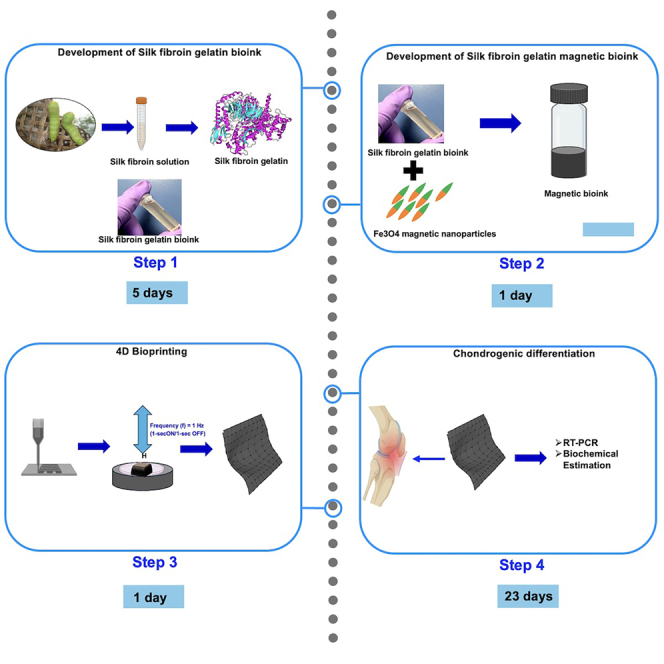

## Teaser

External magnetic fields can regulate cellular responses. Here, we present a protocol to fabricate magnetic constructs by 4D bioprinting with shape-morphing properties using silk fibroin-gelatin bioink for articular cartilage regeneration. We illustrate the steps for magnetic bioink formulation, bioprinting, and chondrogenic induction of human bone marrow mesenchymal stromal cells. We detail the steps to actuate the constructs using an external magnetic field and then characterize chondrogenesis. Magnetic field actuation may be helpful for mechanically activating constructs for articular cartilage regeneration.

## Before you begin

This protocol demonstrates the specific steps in fabricating shape-morphing magnetic constructs using a 4D Bioprinting technique. We have encapsulated novel anisotropic Fe_3_O_4_ magnetic nanoparticles (MNP)^2^ in silk fibroin-gelatin (SF-G) bioink and primed them with human bone marrow mesenchymal stem/stromal cells (BM-MSCs) to ameliorate chondrogenesis of articular cartilage. We illustrate the cyclic actuation of the constructs for 5 and 30 min every other day using an external magnetic field. Molecular characterization of the constructs exhibits superior chondrogenesis when actuated for longer time. Anisotropic novel MNPs were synthesized, as reported previously.^2^

### Cell culture and expansion of human bone marrow mesenchymal stromal cells (BM-MSCs)

**Timing: 3–5 days**

1. Human BM-MSCs culture and passaging.
  a. Seed 5 × 10^5^ cells in a T75 flask with an expansion medium and incubate it in a 5% CO_2_-humified incubator.
  b. Change culture media every 2 days.
  c. When the cells attain 80% confluency, gently aspirate the media from the culture flask to prepare the cells for passaging using trypsin-EDTA.
  d. Post trypsinization, resuspend the cells in 1 mL of fresh expansion media.
  e. Use a hemocytometer or cell counter to determine the cell density.

***Note:*** This stage allows you to verify the cells' concentration, number, and viability

**CRITICAL:** BM-MSCs with passages 3–6 are advised for use owing to their superior proliferative capacity.^3^

### Preparation of media

**Timing: 1 day**

2. Expansion Media.
  a. Add 10% i.e., 50 mL of fetal bovine serum (FBS) to 440 mL of α-MEM with GlutaMAX medium.
  b. To it add 5 mL of 37.5 μg/mL L-AA 2-P, followed by 5 mL of 100 U/mL penicillin-streptomycin.
  c. Mix the constituents properly with a 25 mL serological pipette.
  d. Aliqout the expansion media into aliquots of 50 mL each.
  e. Sterile filter the medium with 0.2 μ syringe filter.
3. Chondrogenic media.
  a. Add 5 mL of L-AA 2-P to 479.45 mL of DMEM/F12 with GlutaMAX medium.
  b. To it add 5 mL of ITS Liquid Media Supplement (100×) and 5 mL of Proline.
  c. Add 5 mL of Penicillin-Streptomycin (10,000 U/mL) to the above mixture.
  d. Mix the constituents well with a 25 mL serological pipette.
  e. Make aliquots of 49.95 mL each in 50 mL conical tubes.
  f. Add 5 μL of Transforming growth factor beta (TGF-β1) to the aliquots freshly before use.
  g. Sterile filter the medium with 0.2 μ syringe filter.

## Key resources table

**Table:** 

REAGENT or RESOURCE	SOURCE	IDENTIFIER
**Chemicals, peptides, and recombinant proteins**		

MEM α with GlutaMAX	Gibco	Cat#32561-029
DMEM/F12 (Gibco) with GlutaMAX	Gibco	Cat#10565018
Fetal bovine serum	Sigma-Aldrich	Cat#F7524
L-ascorbic acid 2-phosphate (L-AA 2-P)	Sigma-Aldrich	Cat#A-8960
Penicillin-streptomycin	Gibco	Cat#15140-122
ITS liquid media supplement (100×)	Sigma-Aldrich	Cat#I3146
Dexamethasone	Sigma-Aldrich	Cat#D-8893
Proline	Sigma-Aldrich	Cat#D8893
Transforming growth factor beta 1 (TGF-β1)	PeproTech	Cat#100-21
Silk cocoons	Central Silk Board, Bangalore, India	N/A
Sodium carbonate	Sigma-Aldrich	Cat#45161
Lithium bromide	Sigma-Aldrich	Cat#213225
Slide-A-Lyzer dialysis cassettes, 3.5 K MWCO	Thermo Fisher Scientific	Cat#66330
Gelatin from porcine skin type A	Sigma-Aldrich	Cat#9000-70-8
Mushroom tyrosinase	Sigma-Aldrich	Cat#T3824-50KU
Protease XIV enzyme	Sigma-Aldrich	Cat#9036-06-0
RNeasy Minikit with on-column DNase treatment	QIAGEN	Cat#74104
iScript cDNA synthesis kit	Bio-Rad	Cat#1708891
SsoAdvanced PreAmp supermix	Bio-Rad	Cat#1725160
iQ SYBR Green supermix	Bio-Rad	Cat#1708880
Proteinase K	Sigma-Aldrich	Cat#P6556
CyQUANT cell proliferation assay kit	Thermo Fisher Scientific	Cat#C7026
Dimethylmethylene blue (DMMB)	Sigma-Aldrich	Cat#341088
Shark cartilage-derived chondroitin sulfate	Sigma-Aldrich	Cat#9082-07-9
Dimethylaminobenzaldehyde reagent	Sigma-Aldrich	Cat#100-10-7

**Biological samples**		

Human BM-MSCs	Texas A&M Health Science Center (Donor d8011L, female, age 22)	Chakraborty et al.^1^

**Other**		

Intertec electromagnet magnetic (1,900 N, 12 V DC, Conrad)	Conrad	Cat#ITS-PE-8245-24VDC
Relay timer circuit	SONGLE R	Type# SRD-12VDC-SL-
Anisotropic Fe_3_O_4_ magnetic nanoparticles (MNPs)	In-house synthesized^2^	N/A
Syringe barrels	Nordson	Cat#7012074
Nordson EFD tapered tip (TT), 27 GA	Nordson	Cat#7018417
1.5 mL Eppendorf tube	Eppendorf	Cat#0030125150
2.0 mL Eppendorf tube	Eppendorf	Cat#0030120094
Ultrasonicator	LABMAN	Model no#LMUC6
RT2 basic hotplate stirrer	Thermo Fisher Scientific	Cat#88880003
Two 2 L beaker	N/A	N/A
Glass rod	N/A	N/A
Aluminum foil	N/A	N/A
Ice cubes	N/A	N/A
Muslin cloth	N/A	N/A
Scissors	N/A	N/A

**Oligonucleotides**		

Glyceraldehyde-3-phosphate-dehydrogenase (GAPDH)	F-ATGGGGAAGGTGAAGGTC G R- TAAAAGCAGCCCTGGTGACC	N/A
Collagen type II alpha 1 chain (Col 2A1)	F-GGCAATAGCAGGTTCACGTACA R-CGATAACAGTCTTGCCCCACTT	N/A
Collagen type I alpha 1 chain (Col 1A1)	F-AGGGCCAAGACGAAGACATC R- AGATCACGTCATCGCACAACA	N/A
Collagen type X alpha 1 chain (Col 10A1)	F-GACTCCCTCCTCACTGTCGC R-AGGGAAGTCTCCCTCACTTGT	N/A
Aggrecan (ACAN)	F-AGGCAGCGTGATCCTTACC R-GGCCTCTCCAGTCTCATTCTC	N/A
SRY-Box Transcription Factor 9 (SOX 9)	F-TTCCGCGACGTGGACAT R-TCAAACTCGTTGACATCGAAGGT	N/A
Matrix metallopeptidase 13 (MMP 13)	F-CCAGACTTCACGATGGCATTG R-GGCATCTCCTCCATAATTTGGC	N/A

## Materials and equipment

### Recipes

**Table:** Expansion medium

Reagent	Final concentration	Amount
Fetal Bovine Serum	10%	50 mL
L-Ascorbic Acid 2-phosphate (L-AA 2-P)	37.5 μg/mL	5 mL
penicillin-streptomycin (10,000 U/mL)	100 U/ mL	5 mL
α-MEM with GlutaMAX	N/A	440 mL
**Total**	**N/A**	**500 mL**

***Note:*** Keep the media at 4°C and utilize it within two weeks.

**Table:** Chondrogenic medium

Reagent	Final concentration	Amount
L-Ascorbic Acid 2-phosphate (L-AA 2-P)	0.1 mM	5 mL
ITS Liquid Media Supplement (100×)	1×	5 mL
Proline	40 μg/ mL	5 mL
Dexamethasone	0.1 μM	500 μL
Penicillin-Streptomycin (10,000 U/mL)	100 U/mL	5 mL
Transforming growth factor beta (TGF-β1)	10 ng/ mL	50 μL
DMEM/F12 with GlutaMAX	N/A	479.45 mL
**Total**	**N/A**	**500 mL**

***Note:*** Keep the media at 4°C and utilize it within two weeks. It’s advisable to add TGF-β1 freshly every time before use.

## Step-by-step method details

### Extraction of silk fibroin (SF) solution

**Timing: 4 days**

**Timing: 4–4.5 h active, overnight drying (day 1) (for step 1)**

**Timing: 4.5 h (day 2) (for step 2)**

**Timing: 49 h (days 2–4) (for step 3)**

**Figure 1:**
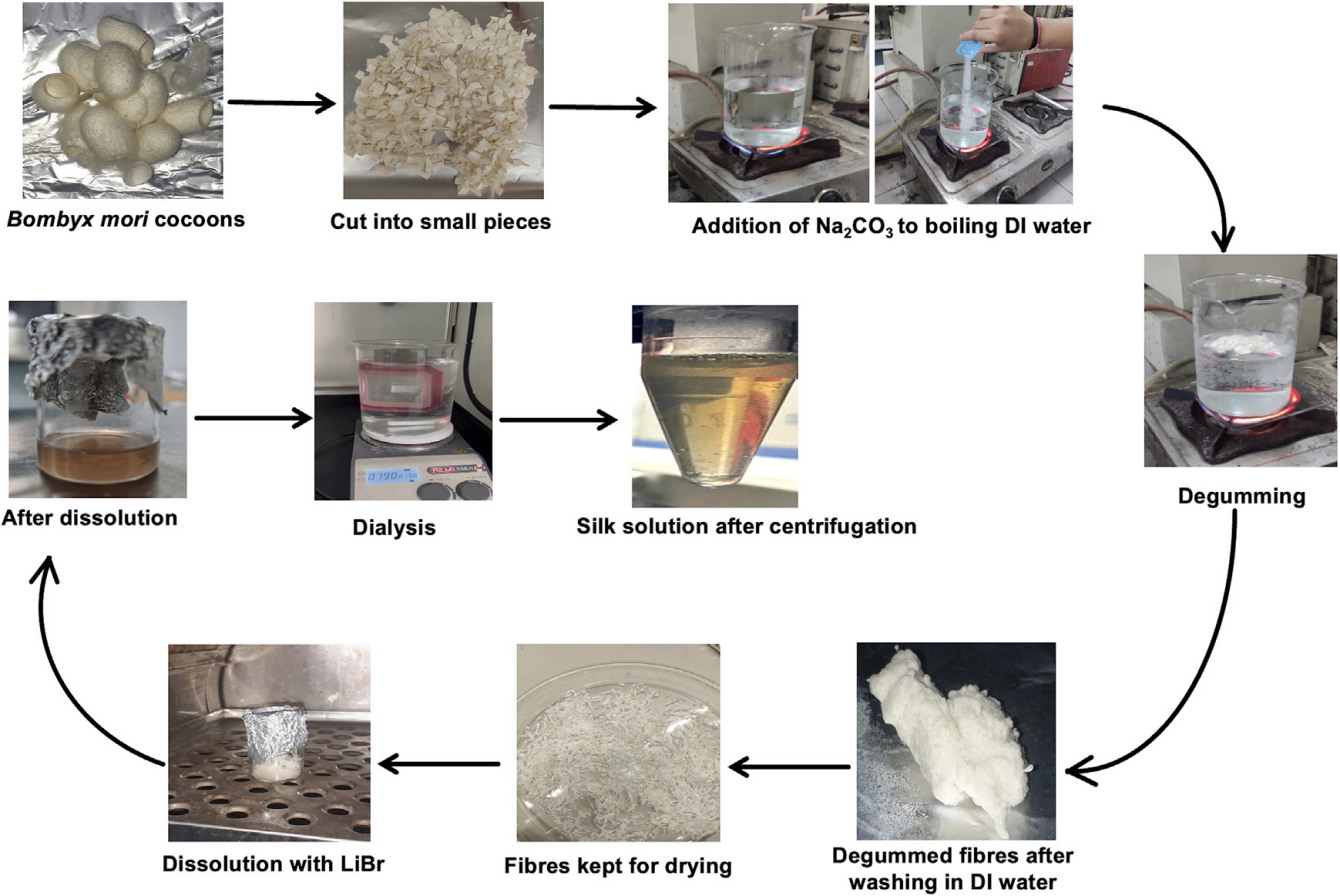
Steps involved in the isolation of silk fibroin solution from *Bombyx mori* cocoons

This step involves the isolation of SF solution from *Bombyx mori* cocoons. The process described previously^4^^,^^5^ was followed to extract the SF solution (Figure 1).

1. Degumming.
  a. Cut silk cocoons with scissors into dime-sized pieces. Measure out 5 g of cocoon pieces into a large weigh boat.
  b. Prepare a 2 L glass beaker filled with 1.5 L of ultrapure water, cover it with aluminum foil and heat until boiling.
    **CRITICAL:** Do not leave the beaker unattended while heating and boiling. Because of high temperatures, plastic beakers should not be used.
  c. Measure 4.24 g of sodium carbonate (Na_2_CO_3_) in a medium weigh boat.
  d. Add the measured Na_2_CO_3_ to the water and let it completely dissolve (to prepare a 0.02 M solution of Na_2_CO_3_).
    **CRITICAL:** If water is boiling, add Na_2_CO_3_ slowly to avoid boiling over.
  e. Add the cocoon pieces once the water starts to boil and continue boiling for 20 min.
    ***Note:*** Occasionally, stir with a glass rod to promote good dispersion of the fibers (poke the fibers do not whirl), meanwhile keep another beaker with ultrapure water ready for boiling.
    **CRITICAL:** To increase reproducibility, boil for exactly 20 min every time. Increasing the boiling time will degrade the fibroin.
  f. Remove the silk fibers with a spatula and cool it by rinsing in ultrapure cold water.
    i. Squeeze excess water out of the fibers using a muslin cloth.
    ii. Discard the Na_2_CO_3_ solution in the sink.
  g. Repeat steps c-e for the second round with the cocoons obtained from step f.
    **CRITICAL:** Degummed fibers and the water used for degumming will be hot, use hand protectors.
  h. Place the degummed fibers in a 1 L beaker filled with 500–600 mL of ultrapure water and a stir bar.
  i. Rinse the fibers in water for 10–15 min on a stir plate, stirring gently.
  j. Repeat Steps h and I around 5–6 times unless the soapy touch is gone.
  k. After the last wash, remove the fibers, squeeze it well with the help of muslin cloth and then spread it out on a clean piece of aluminum foil.
  l. Allow the degummed fibers to dry in a fume hood overnight.
    **Pause point:** Degummed silk fibers, in which the sericin has been removed, can be stored indefinitely at room temperature. For long-term storage, place it in a clean plastic bag or wrap it in aluminum foil. Be sure to indicate the length of the boiling step on the label.
2. Dissolution of degummed fibers in LiBr.
  a. Calculate the amount of 9.3 M lithium bromide (LiBr) needed to prepare a 20% (wt/vol) solution based on the amount of dried fibroin available (e.g., say we have 5 g of degummed silk fibers).
     Making of 20% solution w/v in ultrapure water (uw),
     1 g= (100/20) uw.
     So, 5 g = 5∗5 = 25 mL.
  b. Prepare a 9.3 M LiBr solution.
    (86.85g/mol)(9.3mol/L)(11/1000mL)(X)=…………gofLiBr
    X=(9.3∗86.5∗25)/1000=20.11gofLiBr
    **CRITICAL:** Adding LiBr to water results in an exothermic reaction; be mindful of the heat generated. When preparing large volumes, we recommend carrying this out on ice.
    **CRITICAL:** LiBr has a low density, and its volume should be considered while preparing the solution. We suggest adding only 60% of the calculated volume of water and then bringing the solution up to the final volume. Stir with a small stir bar.
  c. After drying, pack tightly the degummed fibers into a 50 mL glass beaker and add the required amount (as per example = 20.11 g) of LiBr solution on top.
    **CRITICAL:** LiBr must be added to the degummed fibers than adding fibers to the LiBr, so that the fibers will eventually be covered and dissolved by the LiBr. It is also helpful to use the smallest glass container that will still hold the degummed silk fibers and LiBr solution.
  d. Let the degummed silk fibers dissolve in an oven at 60°C for 4 h.
    ***Note:*** Once the fibers are completely dissolved, it will appear amber in color and will be transparent. Black bits from the silkworm may be visible but will be removed later. This solution will be highly viscous, but should not contain any intact fibers, as determined by visual assessment.
3. Dialysis and centrifugation.
  a. Hydrate dialysis cassettes in water for a few minutes.
  b. With a 20 mL syringe and an 18 gauge needle, insert the silk-LiBr solution immediately after dissolution into a dialysis cassette.
    **CRITICAL:** Be careful not to puncture or touch the dialysis membrane. The solution will be very viscous, and this step will be easier if the solution is kept warm (45°C–60°C) before adding to the cassette. It is important to avoid shearing the solution whenever possible to avoid the induction of β-sheet within the silk. Therefore, only use the needle when injecting into the cassette. Moreover, have an additional needle and insert it into another top port of the cassette to allow air to escape. Remove the extra needle once all the air has been purged.
  c. Dialyze against 1 L of cold ultrapure water (add ice cubes) per 12 mL cassette. To ensure mixing, use a large stir bar and place on a magnetic stir plate. Change the water after:
    i. At interval of 1 h each for three times.
    ii. 2 h.
    iii. During evening overnight.
    iv. The next morning.
    v. Afternoon.
    vi. Night.
    vii. As well as in the morning on the following day (i.e., nine changes within 48 h).
  d. Remove silk from the cassettes with another 20 mL syringe and an 18-gauge needle.
    i. Place silk in a 50 mL falcon tube.
    ii. Depending on the volume, either split it between two tubes (if more than 40 mL) or fill one tube and use a counterbalance of water.
  e. Centrifuge to remove impurities. Place in a centrifuge and spin at 9,000 rpm. (∼12,700 G) at 4°C for 20 min.
  f. Carefully remove tubes from the centrifuge and either pour or transfer the silk solution with a 25 mL pipette into another centrifuge tube. Be sure to leave any white flocculent or brown matter behind.
  g. To determine the concentration of the silk in solution.
    i. Measure the weight of a small weigh boat.
    ii. Thereafter, add 0.5 mL of the silk solution to the boat and allow it to dry at 60°C.
    iii. Once the silk is dry, determine the weight of the silk and divide it by 0.5 mL. This will yield the weight per volume percentage.
      **CRITICAL:** Usually, 25 mL of 4%–6% (wt/vol) SF solution can be obtained from a batch of 5 g silk cocoons. Despite having a yellow tinge, the solution should be relatively transparent and have a somewhat higher viscosity than water. It’s recommended that a centrifuge is performed to eliminate any contaminants, such as white flocculants or black particles.
      **Pause point:** The SF solution can be stored at 4°C for at least one month. The amount of time it takes for stored SF solution to gel will vary depending on its purity. Once gelation occurs, another batch of the SF solution must be extracted because it cannot be utilized for further experimentation.

### Maintenance of human BM-MSCs

**Timing: 7 days**

This stage aims to expand the cell population of human BM-MSCs while preserving their stem cell properties and proper morphology by cultivating them till passage 5. Following adequate seeding density and confluency is essential to maintain cell genotype and phenotype during passaging. Post proliferation, the cells will be seeded onto flasks featuring flexible culture surfaces using the standard protocol. By doing this, the cell population is expanded for further experimental studies.

4. Seed 5 × 10^5^ cells in a T75 flask with an expansion medium as described in materials and equipment section and incubate in a 5% CO_2_ humified incubator.
5. Change culture media every 2 days.
6. Gently aspirate the media from the culture flask when the cells attain 80% confluency.
7. Rinse with PBS and add 4 mL of warm 0.05% Trypsin-EDTA. Let it sit at 37°C for 3 min.
8. Add an equal volume of warm expansion media to deactivate the trypsin.
9. Centrifuge in a 15 mL Eppendorf tube at 200 *g* for 3 min at 4°C.
10. Discard the supernatant and resuspend the cell pellet in 1 mL of fresh expansion media.
11. Count the cells using a hemocytometer.

***Note:*** This stage allows you to verify the cells’ concentration, number, and viability

12. 1 × 10^7^ cells are required to prepare the bioink in the subsequent steps.

***Note:*** It’s advisable to use lower passages of BM-MSCs since higher passages modify phenotypic characteristics that may impact the reproducibility of experiments. Moreover, prolonged culture of BM-MSCs should be avoided since this results in cell senescence.^6^^,^^7^

### Preparation of the bioink

**Timing: 4–5 days**

This step details the process involved in the synthesis of MNPs. Further, it describes the process involved in the synthesis of the control and the magnetic bioink.

13. MNPs Synthesis.
  a. To synthesize Fe_2_O_3_ NPs, mix 92.4 g Fe(ClO_4_)_3_.6H_2_O, 1.3 g NaH_2_PO_4_.H_2_O, and 12 g (NH_2_)_2_CO in 2 L of milli-Q water, in a Duran bottle.
  b. Transfer the bottle to an oven preheated at 100°C and leave sealed and undisturbed for 24 h.
  c. Remove the bottle from the oven and allow to cool down at room temperature.
  d. Decant most of the supernatant and transfer the sediment with remaining liquid in 250 mL high-centrifuge tubes.
  e. Centrifuge at 10,000 rpm for 10 min.
  f. Discard supernatant and gently redisperse sedimented particles in fresh milli-Q.
  g. Repeat steps (e-f) at least 4 times, until the supernatant is clear.
  h. After the last washing cycle, discard supernatant and transfer the high-centrifuge tubes in a vacuum oven preheated at 80°C. Leave the tubes in the oven unsealed, under vacuum overnight (at least 16 h).
  i. After the drying cycle, remove tubes from the oven and allow to cool to room temperature under a fume hood.
  j. Remove the solid sediment from the bottom of the tubes using a spatula and transfer to a mortar.
  k. Grind the solid pieces to a fine powder using a pestle and transfer to a sealed, glass vial.
  l. Under a fume hood, transfer the desired mass of particles in a semi-cylindric, ceramic holder and add the required mass of TiH_2_ powder to allow the stoichiometric reduction of Fe_2_O_3_ with H_2_.
  m. Place the holder in a quartz cylinder in a tube furnace and seal the two sides. Connect one side of the cylinder to an argon flow line.
  n. Increase temperature of furnace to 250°C under argon flow. Keep the temperature constant for 5 min under flow to ensure that any traces of moisture have been eliminated.
  o. Close the valves of the tube and stop the argon flow.
  p. Increase furnace temperature to 370°C and keep it constant for 6 h.
  q. Allow furnace temperature to drop to room temperature.
  r. Degas the tube by opening the valves and remove the ceramic holder.
  s. Collect the reduced, black powder (Fe_3_O_4_) and store in a sealed, glass vial.
14. Preparation of the control bioink (SF-G) (Figure 2).
  a. Sterilize gelatin (G) in molecular-grade ethanol overnight inside a laminar flow hood in a 90 mm petridish.
  b. Weigh 8% of G, i.e., 80 mg, under sterile conditions.
  c. In a 2 mL Eppendorf tube, add 8% sterile G to 800 μL of the autoclaved SF (5%) solution. Incubate it at 37°C for 30 min in a water bath.
  d. Add 100 μL of 10X media supplement to the Eppendorf tube.
  e. Add 10% FBS to produce the control acellular bioink.
  f. Suspend 1 × 10^7^ cells/ mL to the acellular control bioink to form the control bioink (SF-G).

**Figure 2:**
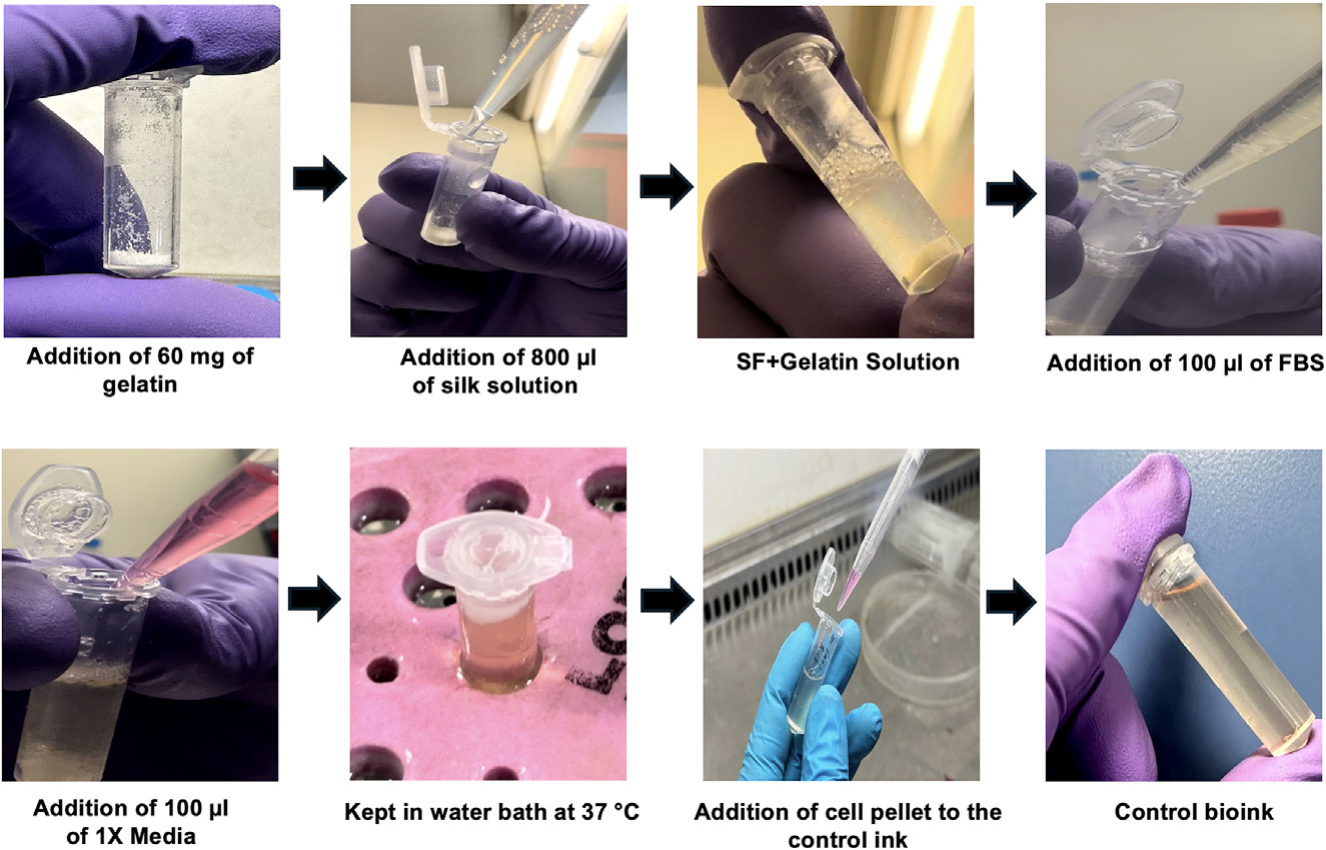
Steps involved in the preparation of the control bioink (SF-G)
15. Preparation of the magnetic bioink (SF-G-MNP) (Figure 3).
  a. Cleanse the MNPs with deionized water 2–3 times. These are morphologically elongated, having an aspect ratio of 3.3 ± 0.5, with an average length of 178 ± 16 nm and a width of 55 ± 7 nm.
  b. Dry them in an oven at 100°C until completely devoid of water.
  c. Sterilize the MNPs for 1 h inside the laminar flow hood with a 90 mm petridish while exposed to UV light.
  d. Ultrasonicate 5 mg of the MNP in 500 μL of PBS for 5 min at 50% amplitude, frequency 20 kHz.
  e. Add the MNP suspension to the control acellular ink.
  f. Adjust the volume to form 5 mg/mL of the MNP containing acellular bioink.
  g. Suspend 1 × 10^7^ cells/mL to the acellular MNP containing acellular bioink to form the SF-G-MNP bioink.

**Figure 3:**
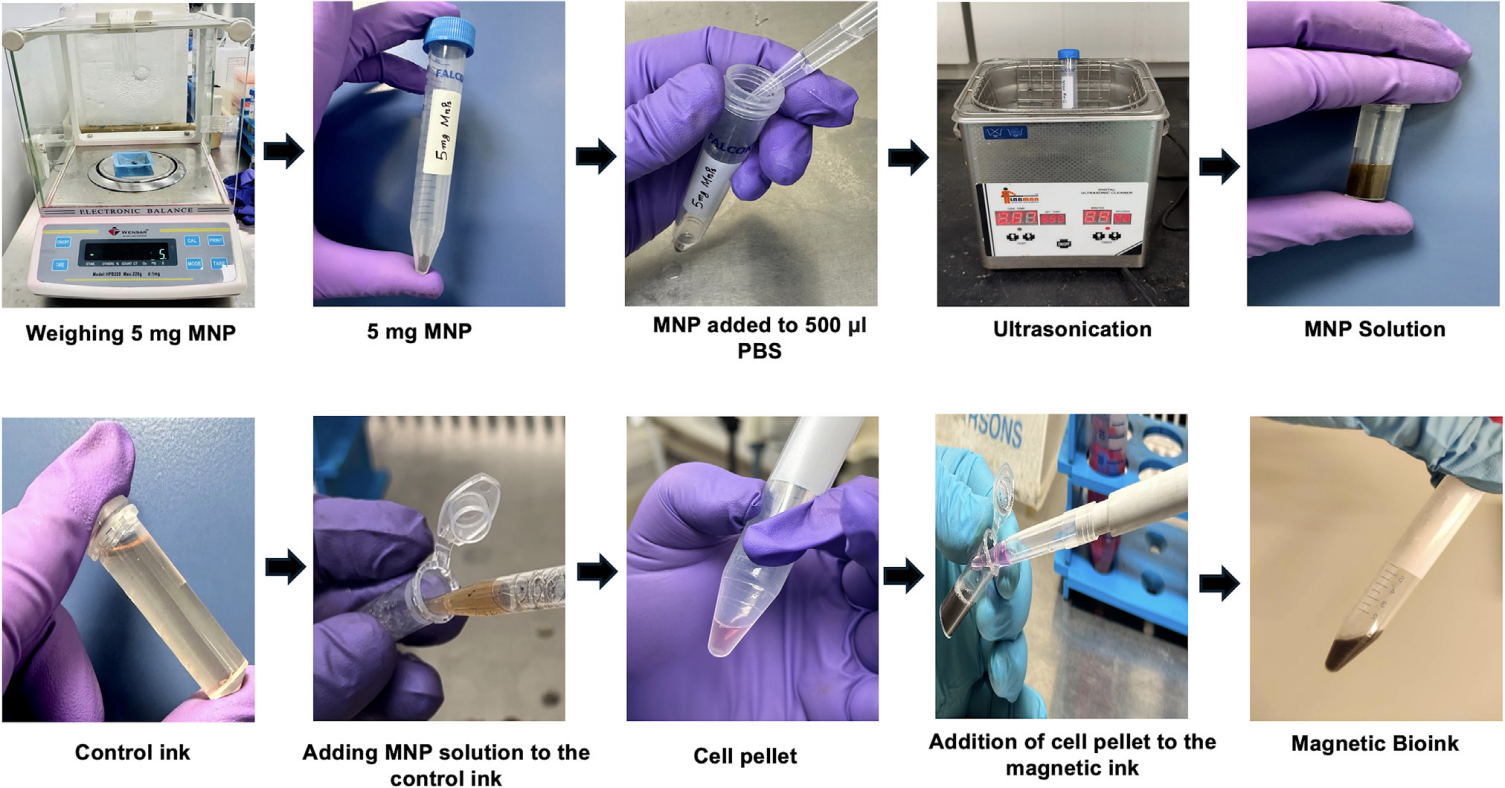
Steps involved in the preparation of the magnetic bioink (SF-G-MNP)

***Note:*** Remove the lid of the petridish while sterilizing the G with ethanol to allow the evaporation of ethanol. Ensure that the G is completely dried before use in bioink formation.

10X media and FBS should be filter sterilized before addition to the SF-G suspension. Acellular control and MNP containing bioink can be prepared a day before and stored at 4°C. However, it is advisable to prepare them fresh before bioprinting.

**CRITICAL:** Addition of SF solution to G often results in the aggregation of the G. Hence, it is advisable to distribute the G along the wall of the 2 mL Eppendorf tube in a single line and then add the SF solution to it by holding the tube at 45°–50° angle.

### Fabrication of the 4D bioprinted construct

**Timing: 1 day (for step 16)**

**Timing: 23 days (for step 17)**

**Timing: 21 days (for step 18)**

16. 3D Bioprinting of the magnetic constructs.
  This step describes the process involved in the fabrication of both the control and magnetic bioprinted construct, followed by their differentiation into chondrogenic lineage. Further, the steps involved in the magnetic actuation of the bioprinted constructs in the presence of an external magnetic field has also been explained.
  a. Sterilize the printing area of the GESIM Bioscaffolder 3.1 printer by turning on the UV of the laminar flow hood for 15–20 min.
    ***Note:*** Before beginning the bioprinting process, all materials such as bioink, syringes, pistons, and needles should be well-dried, sterile, and cleaned with 70% ethanol. Once the biosafety cabinet is closed and the flow is partially on, place these pieces in a large, sterile petri dish and put them under a laminar flow for the entire night to dry them out.
  b. Turn on the bioprinter and double click on the software GESIM Robotics.
  c. Initialize the system by connecting to the printer. It shows the message ‘establishing connection’ and can take more than 10 min to reboot the system.
  d. Upload the configuration.
    ***Note:*** During initialization of the printer after step 16d, verify that every tick that appears is a green “√”. If not, determine the issue and then only move to the next step.
  e. In the target tray option, select the type of tray to use, e.g., MTP6 horizontal for 6 well plate. Calibrate the bottom of the well plate with the Z-sensor.
  f. To calibrate the needle length, select the option measure tool followed by tip calibration and a slow approach.
  g. To calibrate the system’s pressure, select the manual tab option and set the value that must be used (such as 40 kPa, but it depends on the solution).
  h. Once the above parameters are ready, add 800 units mL ^−1^ of mushroom tyrosinase to the control and SF-G-MNP bioink, and pipette gently. Addition of mushroom tyrosinase, induces gelation in the bioink by enzymatic crosslinking.
  i. Add 1 mL of control and SF-G-MNP bioink to a sterile 3 mL syringe separately.
    ***Note:*** The syringe and needle must be perfectly dried; if not, this could interfere with the bioink’s flow and jeopardize cells' viability.
    **CRITICAL:** To remove any air bubbles, centrifuge the syringe containing the bioink by placing a stopper on it. Alternatively, using a positive displacement pipette helps reducing the formation of any air bubble.
  j. Attach the pneumatic piston to the syringe containing bioink.
    **CRITICAL:** Do not use more than 1 mL of bioink since it might result in the bioink's gelation at the bottom of the syringe, which will clog the tip.
  k. Gently mount the syringe onto the dispensing printhead of the bioprinter.
  l. Attach a tapered tip (27 GA) with a nozzle diameter of 210 μm to the syringe.
    **CRITICAL:** It is advisable to use conical nozzles for bioprinting than the blunt ones, since they can significantly decrease the pressure needed and reduce clogging especially with the magnetic bioink.
  m. Set the printing parameters i.e., pressure at 65 ± 20 kPa and speed of 3000 mm/s.
  n. Bioprint the construct (both control and magnetic) with a dimension of 10 mm × 10 mm in length and width, 1.75 mm in height, and 7 layers in non-adherent 6 well plate at 25°C as mentioned in Table 1.
    ***Note:*** Start the bioprinting process 10–15 min after adding mushroom tyrosinase to achieve an initial degree of reticulation.
    **CRITICAL:** Keep enough nozzles, since the nozzles might get clogged during bioprinting.

**Table 1:** Parameters used for Bioprinting

Printing pressure	65 ± 20 kPa
Print temperature	25°C
Print speed	3000 mm/s
No. of layers of the construct	7
Height of the construct	1.75 mm
Length and Breadth of the construct	10 mm × 10 mm
Diameter of the microcapillary nozzle	210 μm
  o. Keep the constructs at room temperature (RT) for 15–20 min (Figure 4).
    **CRITICAL:** Attention should be paid to ensure that the Z-sensor of the Bioscaffolder bioprinter is clean. For this soak a kimwipe with 70% ethanol, which should only touch the tip while cleaning.

**Figure 4:**
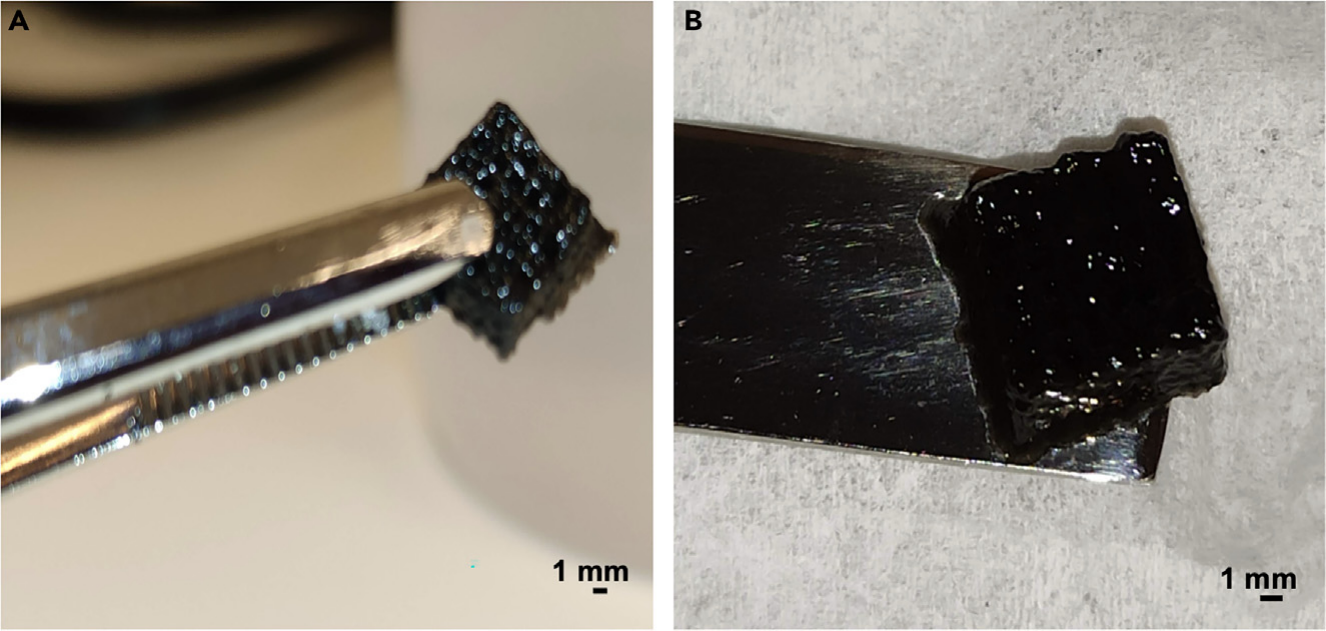
3D Bioprinted magnetic construct (A) after bioprinting (B) after crosslinking. Scale bar: 1 mm.
  Methods video S1. Response of the magnetic construct in presence of an external magnet, related to step 14e
  Methods video S2. Bioprinted constructs change shape when actuated by an external magnetic field, related to step 18e
17. Chondrogenic Differentiation.
  a. After bioprinting add around 2–2.5 mL of expansion media to the constructs kept in a 6 well non adherent plate.
  b. Keep the plates in the incubator for 48 h.
  c. After 48 h, discard the expansion media and add around 2–2.5 mL of chondrogenic media as mentioned in materials and equipment section for chondrogenic differentiation.
  d. Keep the plates inside the incubator and change the media every alternate day for 21 days.
  e. The anisotropic MNP particles allow the constructs to respond in the presence of an external magnet (Methods video S1).
18. Setting up of the Actuation apparatus and magnetic field actuation.
  a. Attach the electromagnet to the circuit board connected to a power supply (Figure 5).

**Figure 5:**
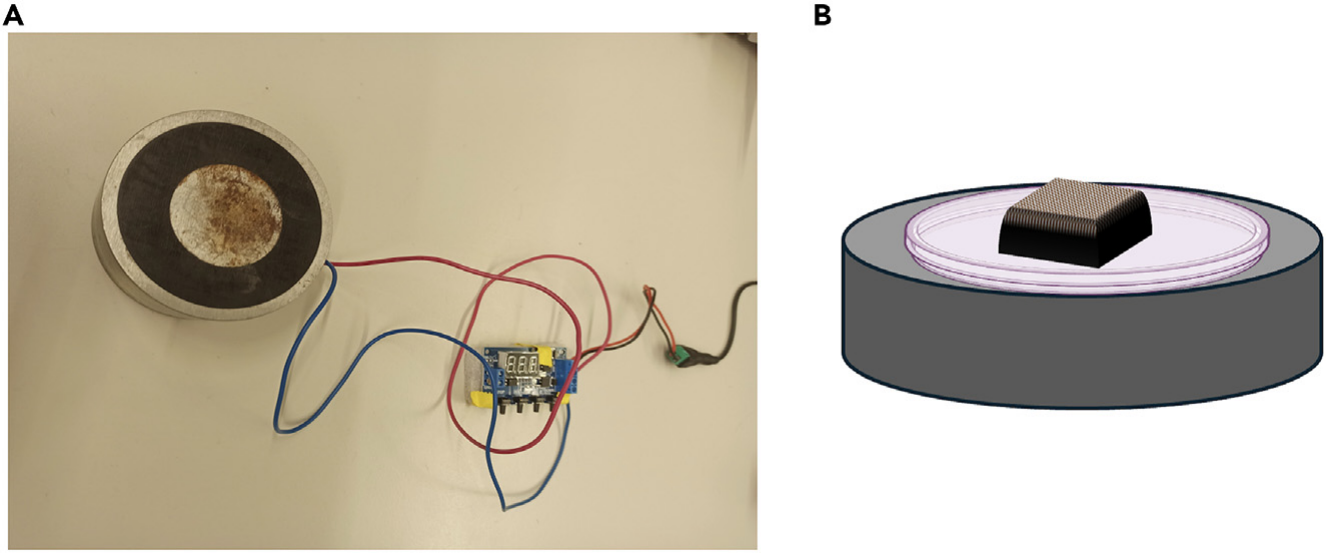
4D Bioprinting of shape morphing constructs (A) The magnetic actuation setup. (B) Exposure of the 4D Bioprinted magnetic construct to an external magnetic field.
  b. Place the construct on the electromagnet in a 35 mm cell culture dish with chondrogenic media.
  c. Divide the constructs into the following groups.
    i. Control: SF-G constructs without any MNP.
    ii. Group A: Actuate magnetic constructs for 5 min every other day.
    iii. Group B: Actuate magnetic constructs for 30 min every other day.
    iv. Group C: Keep magnetic constructs in static condition in the incubator.
  d. Assign the P3.1 function to the circuit board. In order to create a loop, set the trigger key to press on Relay on for 1 s and relay off for 1 s.
  e. Take out Group A and Group B constructs from the incubator and actuate it for 5 min or 30 min in a cyclic ON/OFF pattern by pressing the trigger key of the circuit. This results in shape morphing of the constructs (Methods video S2).
  f. Repeat this every other day maximum for 7 or 21 days.
  g. Keep the control and Group C constructs always inside the incubator.
  h. Collect the constructs on day 7 and 21 for molecular characterization.

**CRITICAL:** Ensure to cool the electromagnet before actuating the next construct, since actuation especially for longer time i.e. Group B constructs, raises the temperature which might be harmful to the cells encapsulated within the construct.

***Note:*** Keep enough backup electric circuits since longer actuation may damage them. Schedule the actuation and media change on the same day so that the constructs are fed with fresh media after the actuation.

### Molecular characterization

**Timing: 5–6 days**

**Timing: 2 days (for step 19)**

**Timing: 2 days (for step 20a)**

**Timing: 1 day (for step 20b)**

**Timing: 2.5 days (for step 20c)**

This step explains the protocol involved in the collection of bioprinted constructs on specific time points, their digestion followed by gene expression analysis. Further the steps involved in DNA, GAG and total collagen estimation have also been detailed briefly.

19. Gene Expression Analysis.
  a. Collect the constructs on day 7 and day 21.
  b. Rinse them with PBS to remove any residual media.
  c. Add 250 μg/ mL of Protease XIV enzyme to the constructs and keep them for 20 min at RT inside the laminar flow hood. This step will induce digestion of the constructs and isolate the cells from the hydrogel.
  d. Place the digested contents in a 15 mL Eppendorf tube and centrifuge it at 1200 rpm to separate the gel debris from the embedded cells.
  e. Following the manufacturer’s instructions (http://www.bea.ki.se/documents/EN-RNeasy%20handbook.pdf), extract total RNA from pellets and reverse transcribe it to cDNA.
  f. Using the chondrogenic marker gene primers or oligonucleotides, perform qPCR.

**Pause point:** Instead of freshly isolating the RNA on days 7 and 21, the constructs can be snap-chilled in liquid nitrogen and stored at −80°C for further analysis. cDNA can also be stored at −80°C for long-term.

***Note:*** Analysis of chondrogenic gene expression is possible throughout the differentiation process at any relevant time point. The sequence of the gene primer can be obtained from site https://pga.mgh.harvard.edu/primerbank/.

20. Biochemical Analysis.
  a. DNA Quantification.
    i. Collect the constructs on day 7 and day 21 in a 1.5 mL Eppendorf tube.
    ii. Instantly snap chill the tubes in liquid nitrogen and store it at −80°C for further analysis.
    iii. Thaw the constructs at RT and place them in 1.5 mL Eppendorf tubes containing 250 μL of 50 mM Tris/1 mM EDTA/1 mM iodoacetamide solution in 1 mg/mL Proteinase K solution.
    iv. Keep the tubes at 56°C for 16 h.
    v. For DNA isolation, free-thaw the samples three times in liquid nitrogen.
    vi. Lyse the samples in lysis buffer (containing RNase A) for 1 h at RT to break down the cellular RNA using the cell proliferation kit.
    vii. To a 96 well plate, add 100 μL of each sample (n = 3) and 100 μL of 2X GR-dye solution and allow it to sit for 15 min at RT.
    viii. Prepare a DNA standard solution and plot a standard curve.
    ix. Measure the fluorescence intensity at 520 nm.
      ***Note:*** The samples from Proteinase K digestion steps can be further utilized to determine GAG and total collagen content.
  b. GAG estimation.
    i. To a 96 well plate, add 25 μL of the proteinase K digested sample.
    ii. Add 150 μL of DMMB solution and 5 μL of 2.3 M NaCl to each well.
    iii. Measure the absorbance at both 525 and 595 nm.
    iv. Determine the absorbance difference.
    v. Prepare the standard curve with chondroitin sulfate derived from shark cartilage.
  c. Total Collagen Estimation using hydroxyproline assay.^1^
    i. Keep the proteinase K digested samples for 18 h hydrolysis at 120°C in the presence of 12 M HCl.
    ii. Dry it for 48 h at 55°C.
    iii. Reconstitute the dried mass in H_2_O.
    iv. Add 100 μL of the Chloramine T/Oxidation buffer mixture to each well (sample and standard), and incubate at RT for 20 min.
    v. Add 100 μL of the diluted DMAB reagent to each well and incubate at 60°C for 20 min.
    vi. Measure the absorbance at 560 nm.
    vii. Correct the readings by subtracting the blank value.
    viii. On the basis of the standard curve, determine the amount of hydroxyproline in the samples.^8^

## Expected outcomes

### Effect of magnetic field actuation on the gene expression profile

On day 21, the Group B constructs, i.e., actuated for 30 min manifested an increase in the expression of the chondrogenic markers, Col 2A1, ACAN, and SOX 9, compared to the control group. These expression levels were higher than the other experimental groups. Group A constructs i.e., actuated for 5 min had expression levels akin to the control, whereas Group C constructs that were kept static showed decreased chondrogenic expression.

**Figure 6:**
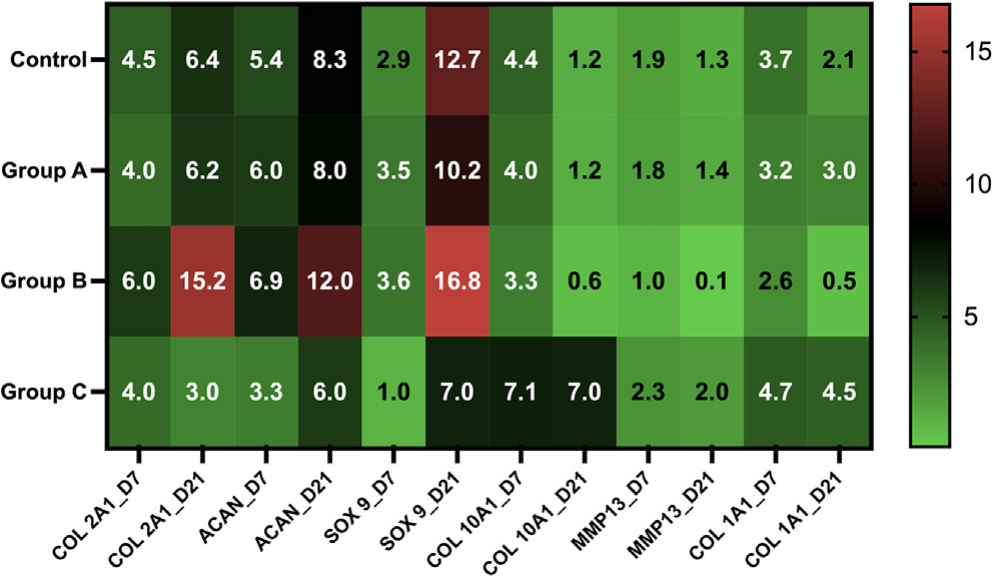
Heat map of RT-PCR data comparing the effect of chondrogenic, hypertrophic and dedifferentiation specific markers at different time points. Figure based on data in Chakraborty et al.^1^ Values refer to the fold increase.

Simultaneously, the Group B constructs i.e., actuated for 30 min exhibited reduced expression for the hypertrophic markers Col 10A1 and MMP 13 in comparison to the other groups. Compared to the expression on day 21, a declining trend was observed for the Group B constructs actuated for 30 min. The expression levels for the de-differentiation marker were similar both on day 7 and day 21 (Figure 6).

### Effect of magnetic field actuation on cell proliferation and ECM synthesis

On day 21, a drop in DNA content was seen in every set of the bioprinted constructs. On the other hand, the Group B constructs when actuated on days 7 and 21 showed maximal DNA content. This was followed by the control, Group B and Group C constructs.

The sulfated GAG was examined to ascertain the chondrogenic differentiation and temporal buildup of the cartilaginous matrix. Day 21 saw increased accumulated GAGs in both the Group A and B and control constructs. In addition, on day 21 as opposed to day 7, the constructs in Group C showed reduced GAG production. When the Group B constructs were actuated on day 21, the highest expression was observed.

**Figure 7:**
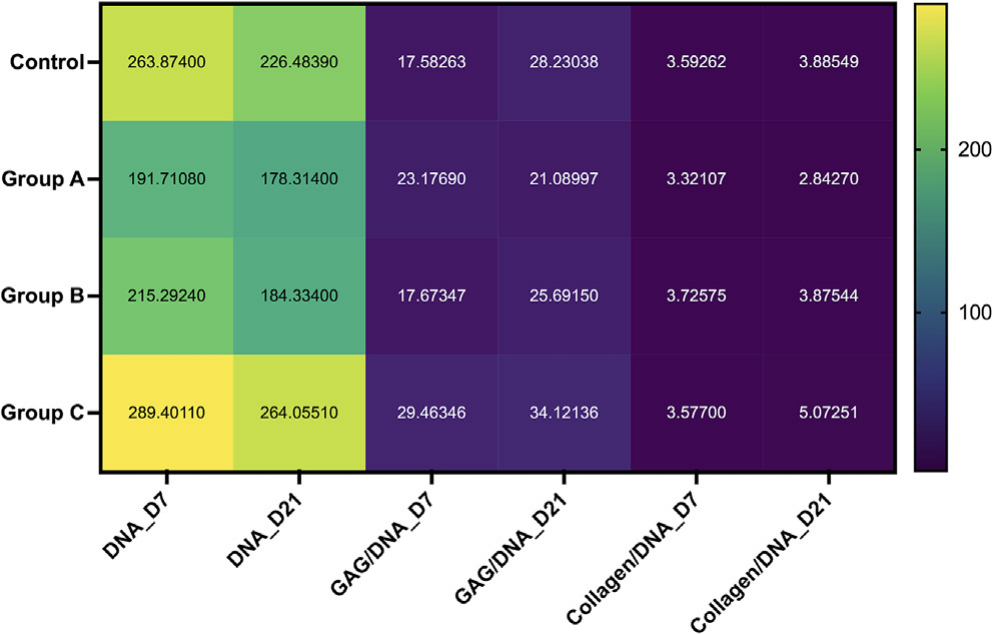
Heat map representing the total DNA content (ng/mL), glycosaminoglycan (GAG) content normalized to the DNA content (ng/ng), and collagen content normalized to the DNA content. Figure based on data in Chakraborty et al.^1^

On days 7 and 21, the amount of extracellular matrix (ECM) formed by the encapsulated human BM-MSCs within the bioprinted constructs was assessed by estimating the total collagen content (Figure 7). On day 21, the Group B constructs showed the highest collagen content. Comparable behavior was displayed by the control samples and the Group A constructs that were actuated for 5 min. On day 21, there was a decrease in the collagen content of the Group C samples.

Interestingly, GAG and collagen estimation normalized to DNA showed a declining tendency with the bioprinted construct in Group C. However, an increasing trend was observed for all the groups on day 21.

## Limitations

While we have extensively demonstrated the rapid efficiency of this protocol in generating shape morphing bioprinted constructs under the effect of magnetic field and using human BM-MSCs, we have not assessed longer term impacts and stability of this method (i.e., more than 4 weeks). We have also not assessed longer stimulation times than 30 min. At the molecular level, although we have found that magnetic actuation for a longer time (30 min) improves the chondrogenic efficiency of the bioprinted constructs compared to when actuated for a shorter time (5 min) or kept static, more detailed mechanism of how these interactions are upregulated still require further investigation. Moreover, the efficiency of our protocol needs to be evaluated with other hydrogel chemistries, as well as in *in vivo* orthotopic cartilage defect models for further analysis and validation. Besides variations over the outcome in terms of chondrogenesis could be expected with different donors.

## Troubleshooting

### Problem 1

Since the MNPs cause the bioink to become more viscous, this results in loss of the bioink while pipetting it into the syringe barrel, as mentioned in step 16i.

### Potential solution

Use of a 1000 μL positive-displacement pipette helps in the smooth pipetting of the viscous bioink without any loss.

### Problem 2

No macroscopically discernible form change occurred during magnetic actuation when using 10% or higher concentration gelatin.

### Potential solution

Optimizing the gelatin concentration i.e., reducing it from higher to lower concentration (8%), results in soft hydrogels that show also macroscopically shape morphing characteristics upon actuation.

## Resource availability

### Lead contact

Further information and requests for resources and reagents should be directed to and will be fulfilled by the lead contact, Lorenzo Moroni (l.moroni@maastrichtuniversity.nl).

### Technical contact

Technical questions on executing this protocol should be directed to and will be answered by the technical contact, Juhi Chakraborty (chakraborty.juhi21@gmail.com).

### Materials availability

This study did not generate new unique reagents.

### Data and code availability

• This paper does not report any code.
• Any additional information required to reanalyze the data reported in this paper is available from the lead contact upon request.

## Acknowledgments

This work was funded by ZonMw (project number 11630095101). This work is also part of the project 3D-MENTOR (with project number 18647) of the Vici research program, financed by the Dutch Research Council (NWO).

## Author contributions

J.C., methodology, conceptualization, investigation, writing, and review and editing; C.R., methodology; M.K., methodology; C.M., review and editing and funding acquisition; S.G., supervision, review and editing, project administration, and conceptualization; L.M., supervision, review and editing, project administration, conceptualization, and funding acquisition.

## Declaration of interests

The authors declare no competing interests.
